# Supplementary material for: Determination of PaO2/FiO2 after 24 h of invasive mechanical ventilation and ΔPaO2/FiO2 at 24 h as predictors of survival in patients diagnosed with ARDS due to COVID-19
Source: PeerJ. 2022 Dec 13;10:e14290. doi: 10.7717/peerj.14290 (PMC9756861; doi:10.7717/peerj.14290)
Supplement: Table S1 — * Mean and standard deviation ** Median and interquartile range a Anova b Fisher’s exact c Kruskal Wallis DM2, Type 2 diabetes mellitus, AHT, Arterial hypertension, ICU, Intensive care unit, MV, Mechanical ventilation, SatO2, Oxygen saturation, CRP, C-reactive protein, LDH, Lactate dehydrogenase, CPK, Creatinine phosphokinase, AST, Aspartate aminotransferase, ALT, Alanine aminotransferase. [file peerj-10-14290-s001.docx]

**Supplementary Table 1:** Clinical and laboratory characteristics of the study population and comparison according to the severity of ARDS after 24 hours of MV

| **Variable** | **Mild (n=149)** | **Moderate (n=45)** | **Severe (n=6)** | **p-Value** |
| --- | --- | --- | --- | --- |
| Demographic characteristics |  |  |  |  |
| Age (years)* | 52.94 (±12.59) | 57.57 (±9.59) | 64 (±13.23) | **0.001 ^a^** |
| - <50 years (%) | 64 (42.95) | 9 (20.0) | 1 (16.67) | **0.003 ^b^** |
| - 50-59 years (%) | 40 (26.85) | 16 (35.56) | 2 (33.33) |  |
| - ≥60 years (%) | 45 (30.20) | 20 (44.44) | 3 (50) |  |
| Sex |  |  |  | 0.723 ^b^ |
| - Female | 30 (20.13) | 10 (22.22) | 2 (33.33) |  |
| - Male | 119 (79.87) | 35 (77.78) | 4 (66.67) |  |
| Number of comorbidities ** | 1 (0-2) | 1 (1-3) | 3 (3-4) | **0.001 ^c^** |
| - No comorbidity (%) | 37 (24.83) | 7 (15.56) | 0 (0) | 0.118 ^b^ |
| - Only 1 | 61 (40.64) | 17 (37.78) | 1 (16.67) |  |
| - More than 2 | 51 (34.23) | 21 (46.67) | 5 (83.33) |  |
| Comorbidities |  |  |  |  |
| - Obesity (%) | 87 (58.39) | 28 (62.22 | 3 (50) | 0.765 ^b^ |
| - DM2 (%) | 32 (21.48) | 10 (22.22) | 1 (16.67) | 0.999 ^b^ |
| - AHT (%) | 35 (23.49) | 14 (31.11) | 4 (66.67) | **0.048** ^b^ |
| -Heart failure | 3 (2.01) | 5 (11.11) | 2 (33.33) | **0.001 ^b^** |
| - Asthma (%) | 19 (12.75) | 8 (17.78) | 3 (50) | 0.051 ^b^ |
| - Chronic kidney disease (%) | 0 (0) | 6 (13.33) | 2 (33.33) | **<0.001 ^b^** |
| - Immunosuppression (%) | 7 (4.73) | 7 (15.56) | 4 (66.67) | **<0.001 ^b^** |
| Clinical and ventilatory characteristics |  |  |  |  |
| - Hospital stay time (days) ** | 20 (14-28) | 21 (17-29) | 8.5 (6-22) | **0.046 ^c^** |
| - Time in ICU (days) ** | 9 (5-14) | 12 (9-19) | 8.5 (2-16) | **0.002 ^c^** |
| - Time in IMV (days) ** | 8 (5-13) | 13 (9-21) | 9 (3-17) | **<0.001 ^c^** |
| - SatO2 (%) ** | 89 (85-91) | 86 (80-89) | 77 (65-86) | **<0.001 ^c^** |
| - PaO2/FiO2 at hospital admission** | 256 (189-306) | 174 (116-260) | 181.5 (60-245) | **<0.001 ^c^** |
| - PaO2/FiO2 before IMV ** | 104 (79.3-136) | 66.5 (56-82) | 54.5 (50-68) | **<0.001 ^c^** |
| - Shock and need for vasopressors (%) | 42 (28.38) | 23 (51.11) | 5 (83.33) | **0.001 ^b^** |
| - Lung damage on CT (%)* | 58.02 (±12.70) | 69.86 (±13.21) | 80 (±10.48) | **0.001 ^a^** |
| - Tidal volume 24h after IMV ** | 466 (440-504) | 450 (400-507) | 478 (320-510) | 0.199 ^c^ |
| - PEEP 24h after IMV ** | 12 (10-14) | 12 (12-14) | 12 (12-13) | 0.178 ^c^ |
| - Inspiratory pressure 24h after IMV ** | 15 (14-17) | 16 (15-20) | 14 (13-16) | **0.042 ^c^** |
| - Plateau pressure 24h after IMV ** | 28 (26-30) | 30 (28-34) | 29 (26-39) | **<0.001 ^c^** |
| - Driving pressure 24h after IMV ** | 16 (14-18) | 18 (16-21) | 17 (15-24) | **0.001 ^c^** |
| - SOFA upon admission to IMV ** | 4 (3-4) | 5 (4-5) | 6 (6-9) | **<0.001 ^c^** |
| Laboratory Characteristics |  |  |  |  |
| - Leukocytes (cells/mm3) ** | 9240 (6950-12070) | 10220 (7770-16170) | 9910 (3540-12880) | 0.295 ^c^ |
| - Lymphocytes (cells/mm3) ** | 686 (488.5-1050) | 642 (480-1062.8) | 441 (283.2-1191.2) | 0.471 ^c^ |
| - Platelets (cells x 10^3^/L) ** | 290 (231-366) | 283 (215-341) | 180 (146-241) | **0.040 ^c^** |
| - CRP (mg/dL) ** | 12.52 (5.35-17.28) | 15 (10.13-20.26) | 14.51 (12.37-19.8) | **0.048 ^c^** |
| - Procalcitonin (ng/mL) ** | 0.12 (0.05-0.25) | 0.3 (0.13-0.81) | 0.45 (0.22-1.86) | **0.003 ^c^** |
| -LDH (U/L) ** | 727 (581-887) | 911 (616-1272) | 1072.5 (982-1489) | **0.001 ^c^** |
| - CPK-Total (U/L) ** | 122 (68-260) | 151 (63-260) | 141.5 (111-225) | 0.810 ^c^ |
| - CPK-MB (U/L) ** | 25.75 (19.9-34.7) | 27.3 (20-48.3) | 31.7 (22.1-35) | 0.491 ^c^ |
| - AST (U/L) ** | 51 (32-79) | 50 (27-81) | 62.5 (28-96) | 0.939 ^c^ |
| - ALT (U/L) ** | 70 (40-133) | 63 (31-108) | 46 (25-105) | 0.151 ^c^ |
| - Total bilirubin (mg/dL) ** | 0.68 (0.5-0.96) | 0.53 (0.38-0.9) | 0.69 (0.39-1.3) | 0.308 ^c^ |
| - Creatinine (mg/dL) ** | 0.82 (0.68-0.95) | 0.89 (0.79-1.1) | 1.26 (1.14-7.33) | **<0.001 ^c^** |

* Mean and standard deviation ** Median and interquartile range ^a^ Anova ^b^ Fisher's exact ^c^ Kruskal Wallis

DM2: Type 2 diabetes mellitus, AHT: Arterial hypertension, ICU: Intensive care unit, MV: Mechanical ventilation, SatO2: Oxygen saturation, CRP: C-reactive protein, LDH: Lactate dehydrogenase, CPK: Creatinine phosphokinase, AST: Aspartate aminotransferase, ALT: Alanine aminotransferase.
